# Experimental Investigation on Bending Behavior of Existing RC Beam Retrofitted with SMA-ECC Composites Materials

**DOI:** 10.3390/ma15010012

**Published:** 2021-12-21

**Authors:** Hui Qian, Qingyuan Zhang, Xun Zhang, Enfeng Deng, Jundong Gao

**Affiliations:** School of Civil Engineering, Zhengzhou University, Zhengzhou 450001, China; qianhui@zzu.edu.cn (H.Q.); dayuanzi815@126.com (Q.Z.); dengenfeng@zzu.edu.cn (E.D.); gaojundong@zzu.edu.cn (J.G.)

**Keywords:** shape memory alloys, engineered cementitious composites, composites materials, self-recovery capacity, bending behavior

## Abstract

In order to realize the self-centering, high energy consumption, and high ductility of the existing building structure through strengthening and retrofit of structure, a method of reinforced concrete (RC) beam strengthened by using Shape Memory Alloy (SMA) and Engineered Cementitious Composites (ECC) was proposed. Four kinds of specimens were designed, including one beam strengthened with enlarging section area of steel reinforced concrete, one beam strengthened with enlarging section area of SMA reinforced concrete, beam strengthened with enlarging section area of SMA reinforced ECC, and beam strengthened with enlarging section area of steel reinforced ECC; these specimens were manufactured for the monotonic cycle loading tests study on its bending behavior. The influence on the bearing capacity, energy dissipation performance, and self-recovery capacity for each test specimens with different strengthening materials were investigated, especially the bending behavior of the beams strengthened by SMA reinforced ECC. The results show that, compared with the ordinary reinforced concrete beams, strengthening existing RC beam with enlarging section area of SMA reinforced ECC can improve the self-recovery capacity, ductility, and deformability of the specimens. Finally, a revised design formula for the bending capacity of RC beams, strengthened with enlarging sections of ECC, was proposed by considering the tensile capacity provided by ECC, and the calculated values are in good agreement with the experimental value, indicating that the revised formula can be well applied to the beam strengthening with enlarging section of SMA-ECC Materials.

## 1. Introduction

At present, among many structural forms in China, reinforced concrete (RC) structure is the most widely used structural type. As the service time of the structure increases, the performance of reinforced concrete will be affected by factors, such as adverse environment, aging, concrete carbonization, etc. [1]. These factors will not only affect the mechanical performance of RC structure, but also threaten the personal safety of the users. Therefore, in order to make existing RC structure meet the requirements of using functions and safety, it is great significance to carry out the research on the strengthening and retrofitting of existing RC structures. In recent years, many scholars have proved that the use of high-performance materials and intelligent structural members can better serve the strengthening and retrofitting of existing RC structures [2].

Shape Memory Alloys (SMA) is a new type of intelligent material with shape memory effect, super-elasticity, high damping, and fatigue resistance. If SMA is used as the longitudinal reinforcement of concrete beam, it can provide good self-recovery capacity for concrete beam. However, due to the high price of SMA, it is rarely used in new structures. It can still be used in the strengthening works of some important structures. Many scholars in the world have carried out a series of research on the self-recovery structural system based on SMA. For example, energy dissipation bracings [3,4], dampers [5,6,7,8], composite isolation bearings [9,10,11,12], energy dissipation coupling beams [13,14], etc. Great progress has been made in such areas. For the seismic performance of structural members, e.g., SMA reinforced beams [15,16,17], SMA reinforced pier columns [18,19,20], SMA reinforced shear walls [21,22] and joints [23,24,25], as well as the structural strengthening and retrofitting technology based on SMA materials [26,27,28], have been studied.

Engineered Cementitious Composites (ECC) is a kind of high-performance cementitious composite with obvious strain hardening characteristics and good crack control ability [29,30]. Ding et al. [31], Wu et al. [32], Yang et al. [33], and Said et al. [34] have carried out the research on beams, columns, walls, joints, and other components casted with ECC, respectively. These studies indicates that, compared with ordinary concrete, ECC has excellent tensile performance, fine cracking mechanism, and good ductility. It can solve various problems in engineering maintenance and strengthening works, such as improving impermeability, crack resistance, structural durability, and so on. ECC can also improve the bearing capacity and seismic performance of those engineering structural members at the same time.

For the composite structure of SMA reinforced ECC, scholars have studied beams [35,36], pier columns [37,38], shear walls [39], joints [40], and other structural members. This research indicates that the combination with SMA and ECC can insure both ECC and SMA in use with their optimal capacity respectively, and thereby satisfy the structural demands.

Enlarged section method is a traditional strengthening method of concrete structure. It is a strengthening method to improve the bearing capacity of original members by increasing section area and reinforcement area. This method can significantly improve the mechanical performance of members because of the increase of member section. However, the component size becomes larger after strengthening, which may affect the serviceability of the structure. Therefore, the premise of this strengthening method is that it does not affect the serviceability of the structure. At present, the strengthening method of concrete structure pasted with FRP has also been widely studied [41,42,43]. Its advantage is that the strength and durability of structural members can be improved without increasing the self-weight of the structure and the member section. However, the fire resistance of FRP is poor, and the fire prevention treatment further increases the cost of strengthening works.

In summary, the durability of concrete can be improved significantly by the super-elasticity of SMA and high toughness and fine cracking mechanism of ECC. Therefore, this paper proposes to use the enlarge section area of SMA reinforced ECC to strengthen the existing RC beams. Four types of strengthened beams were designed and fabricated. Through monotonic cycle loading tests, the influences on the bearing capacity, energy dissipation performance, and self-recovery capacity of the test beams with different strengthening materials are investigated, especially the bending behavior of the beams strengthened by SMA reinforced ECC.

## 2. Test Overview

### 2.1. Specimen Design

**Figure 1:**
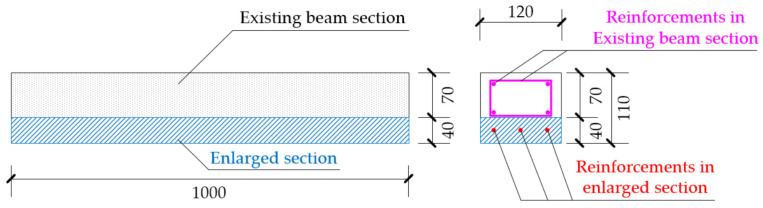
Specifications of the test specimens.

Due to the limitation of the loading capacity of the testing device, the section of the specimen needs to be controlled below then 130 × 130 mm. At the same time, in order to meet the requirements of the minimum thickness of concrete/ECC cover of enlarged section, the height of enlarged section must meet the minimum requirements of 30 mm. Based on the above principles, the member section is determined as: the existing beam length is 1000 mm, and the original beam section is a rectangle of width × height = 120 mm × 80 mm before the strengthening, and the upper and lower reinforcements are 2 HRB355 steel bars with diameter of 6 mm, and the stirrup is HPB300 steel bars with diameter of 6 mm and spacing of 100 mm. Strengthening is carried out after the existing beam has been fully cured. The strengthening method is: firstly, chisel off the 10 mm protective layer at the bottom of the test piece and then roughen the bottom surface; finally, the enlarged section will be poured at the beam bottom by secondary pouring, as shown in Figure 1. The cross section of the beam after strengthening is 120 mm × 110 mm, the enlarged section at the bottom of beam is Δ*h* = 40 mm (including the chiseled 10 mm protective layer). The specifications of the test specimens are shown in Figure 1.

A total of 6 specimens with the same shape and size were produced in this test, of which SJ-1 is strengthened with steel reinforced concrete, SJ-2 is strengthened with SMA reinforced concrete, SJ-3 is strengthened with SMA reinforced ECC, and SJ-4 is strengthened with steel reinforced ECC. SJ-5 and SJ-6 are two spare test pieces, which are designed as the same as SJ-1 and SJ-3, respectively. The reinforcement ratio of enlarged section is designed according to the principle of the same total tensile bearing capacity of reinforcements in the enlarged section. The design parameters of specimens are shown in Table 1.

### 2.2. Material Test of Specimens

#### 2.2.1. Shape Memory Alloy

**Figure 2:**
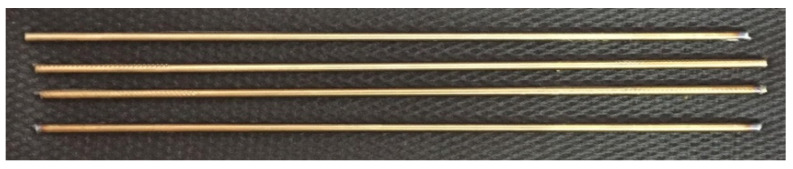
NiTi SMA bars with the diameter of 5.5 mm.

In the material test, the test specimen of SMA bar has a diameter of 5.5 mm, a length of 250 mm, and a gauge length of 150 mm, as shown in Figure 2. The composition of SMA is Ti-56.35at%Ni, and the completion temperature of reverse martensitic transformation (*A*_f_) is −10 °C. After the test piece is processed into an annealed state, it will be heat-treated. The heat treatment process of SMA bar is at 400 °C for 30 min, followed by water quenching [44].

**Figure 3:**
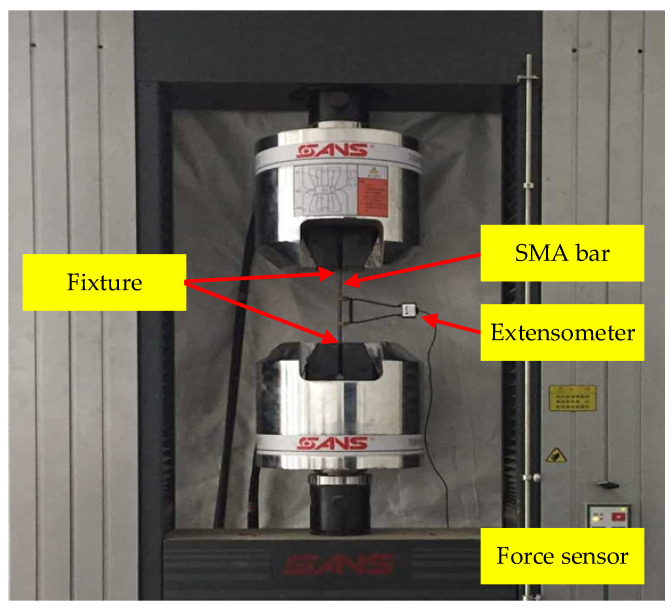
CMT electro-hydraulic servo universal material testing machine.

**Figure 4:**
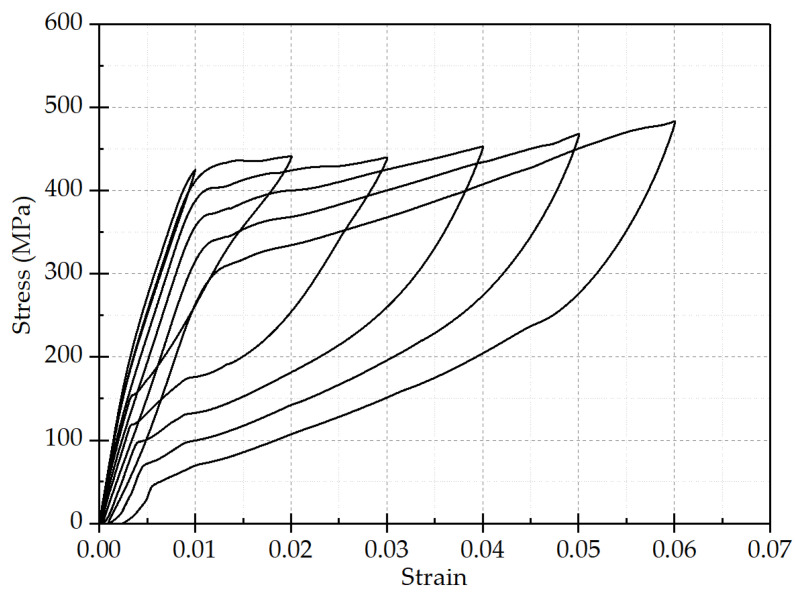
Stress–strain relationship curve of NiTi SMA bars under cyclic tensile load.

The test device adopts the CMT (Crane Motor Traction) electro-hydraulic servo universal material testing machine controlled by a microcomputer, as shown in Figure 3. In order to ensure the stability of the material performance of SMA, the SMA bar should be treated under thermal-cooling cycle treatment before the material test. The thermal-cooling cycle treatment method requires that the SMA bars should be placed in boiling water (100 °C) for 5 min, and then taken out and placed in cold water for 5 min. This treatment method was performed alternately five times before the test. Finally, the test specimens should be taken out from boiling water and cooled naturally at room temperature. The material tensile tests were performed on SMA bars with increasing strain amplitudes as 1%, 2%, 3%, 4%, 5%, 6%, etc. The material tensile tests data are shown in Figure 4.

Through the analysis of the material tensile test results, it can be concluded that:

(1) With the increase of strain amplitude, the phase transformation stress of the hyper-elastic SMA bar gradually decreases, the recovery stress gradually increases, and finally tends to be stable with the decrease of strain amplitude. Therefore, the SMA bar is stretched under circulation of loading and unloading is conducive to the stability of its material properties before it is used.
(2) The phase transformation stress and recovery stress tend to be stable with the increase of loading cycle; the residual strain gradually increases during the loading process, and the variation range becomes smaller and smaller. Therefore, SMA bars are stretched under circulation of loading and unloading before use, which is also conducive to improve the super-elasticity of SMA.
(3) As the strain amplitude increases, the residual strain of SMA gradually increases, and the maximum residual strain is only 0.003, indicating that the SMA bars used in the material tests have good recovery ability. With the increasing loading cycle, the increasing rate of residual strain gradually slows down, and the residual strain tends to be stable.

In summary, monotonic cycle loading can make the mechanical properties of SMA more stable, in order to ensure that the material properties of SMA can be significantly displayed in the tests.

#### 2.2.2. ECC and Ordinary Concrete

**Figure 5:**
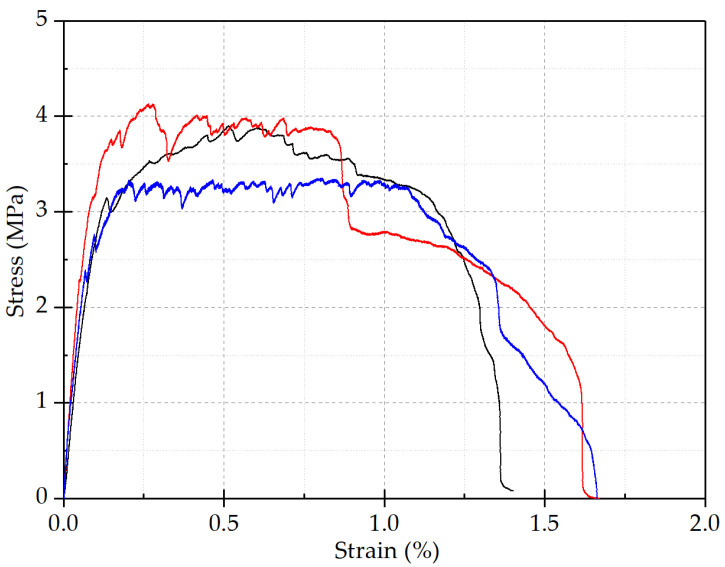
ECC tensile stress–strain curve.

This test uses concrete with a strength grade of C30, and ECC adopts high-strength PVA (polyvinyl alcohol) fiber-reinforced cement mortar. Its components include cement, water, fly ash, fine sand, PVA fiber, and admixtures, which are configured according to the mix proportion given in Table 2. Among them, the content of PVA fiber is 2% by volume, the specification of PVA fiber is A 0.02 × 8, and the tensile strength is 1400 MPa. Tensile tests are performed on 3 ECC test specimens, and the test results are given in Table 3. The average tensile strength of the test pieces was 3.87 MPa, which indicates that ECC has good ductility. ECC tensile stress–strain curve is shown in Figure 5.

### 2.3. Monotonic Cycle Loading Test

#### 2.3.1. Test Device and Loading System

**Figure 6:**
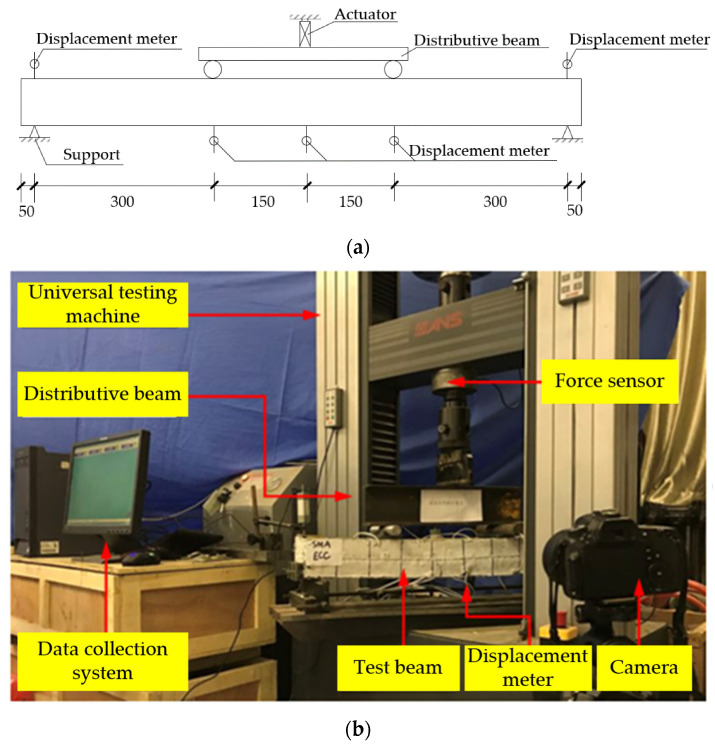
Test setup for quasi-static cyclic tests. (**a**) Sketch of the test loading device. (**b**) Photograph of the test setup.

This test loading device adopts the CMT microcomputer controlled electro-hydraulic servo universal testing machine in the Structure Laboratory of Zhengzhou University, and the measuring range of force sensor is 2000 kN. The test adopts four-point bending loading, and the force were applied to the trisection point of the beam by the distributing beam. All test data are automatically collected by the test software of Power Test V3.4. The loading device is shown in Figure 6. The measuring points of strain of reinforcements are arranged in the middle of the span.

#### 2.3.2. Loading Protocol

**Figure 7:**
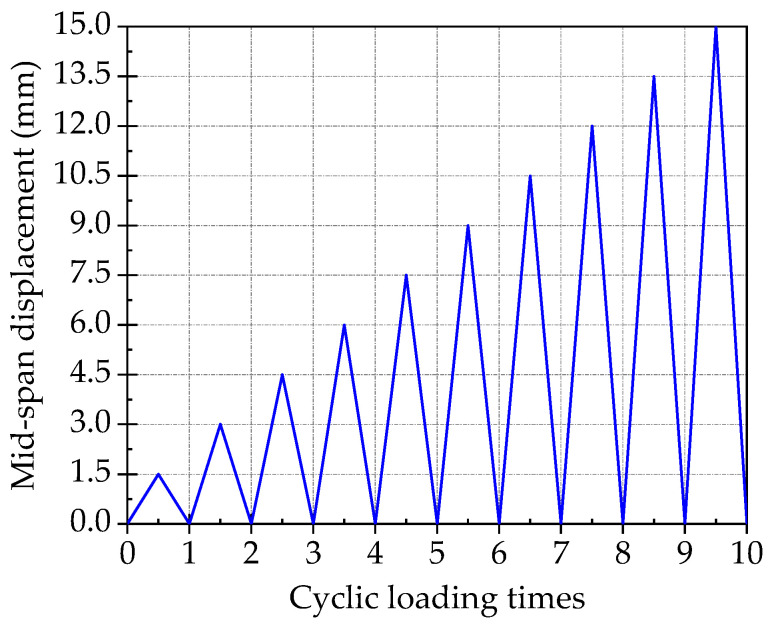
Monotonic cycle loading protocol.

Using variable amplitude displacement control loading mode. Firstly, the test specimen should be preloaded once before the formal loading, in order to check whether the loading equipment and instruments can work normally. The value of preloading should be less than the design cracking load *P*_cr_. Then, the vertical ultimate displacement Δ in the middle of the span should be determined by monotonic loading test on the specimen of SJ-5. The results of monotonic loading test on the specimen of SJ-5 shows that the Δ is about 15 mm. Therefore, in the formal loading, the displacement of the initial loading cycle is 1.5 mm, and then the displacement of each cycle increases by 1.5 mm progressively. The monotonic cycle loading protocol is shown in Figure 7.

## 3. Test Results and Analysis

### 3.1. Failure Process

(1) SJ-1 (Strengthened by Steel reinforced concrete)

**Figure 8:**
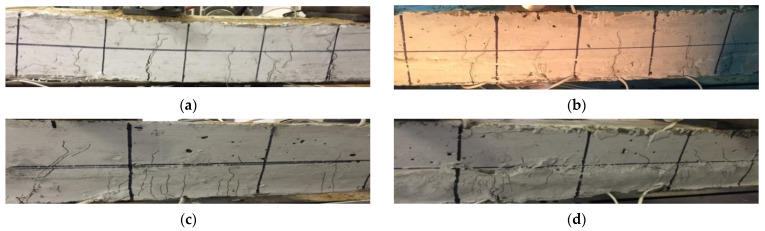
Failure modes of specimens. (**a**) SJ-1; (**b**) SJ-2; (**c**) SJ-3; (**d**) SJ-4.

Six cracks were observed in the pure bending section of the specimen. As the load increased, the width of the cracks increased, and finally part of the concrete was crushed. After unloading, the number of cracks and the crack widths remain basically unchanged, and the ultimate bearing capacity was 32.16 kN. The failure mode of the specimen after unloading is shown in Figure 8a.

(2) SJ-2 (Strengthened by SMA reinforced concrete)

The failure mode of specimen SJ-2 was similar to that of SJ-1, and the number of vertical cracks observed by SJ-2 is slightly reduced. After unloading, the maximum crack width at the beam bottom decreased, some small cracks were closed, and the ultimate bearing capacity was 27.17 kN. The failure mode of the specimen after unloading is shown in Figure 8b.

(3) SJ-3 (Strengthened by SMA reinforced ECC)

**Figure 9:**
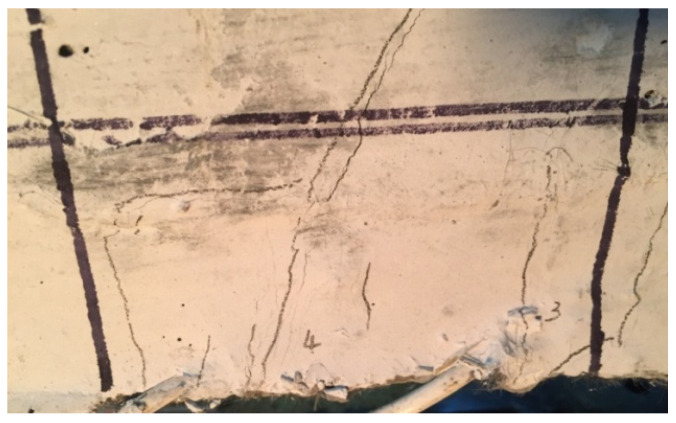
The crack distribution of SJ-3.

Due to the characteristics of ECC, there was no obvious main crack in the strengthened section at the bottom of the beam until the end of loading. In the existing beam section, there were still main cracks that appeared. With the increase of loading cycle, the number of micro-cracks in the strengthened section of the specimen increased, but the width of these cracks increased slowly. Finally, more than 70 micro-cracks were counted in the pure bending section of the strengthened section. The crack distribution of specimen SJ-3 was shown in the Figure 9. After unloading, most of micro-cracks were closed, only 3 cracks were still observed, and the ultimate bearing capacity of the specimen was 25.31 kN. The failure mode of the specimen after unloading is shown in Figure 8c.

(4) SJ-4 (Strengthened by steel reinforced ECC)

ECC material was also used in specimen SJ-4, so the development of cracks during loading was basically similar to SJ-3, mainly a large number of fine micro-cracks. With the increase of the load, the number of micro-cracks in the strengthened section of the specimen increased obviously. Finally, more than 40 micro-cracks were counted in the pure bending section of the strengthened section. The width of the cracks was small, and it appeared as obvious multiple micro-cracking when it failed. After unloading, the crack width was basically unchanged, and the ultimate bearing capacity was 30.16 KN. The failure mode of the specimen after unloading is shown in Figure 8d.

It can be clearly seen from Figure 8 and Figure 9 that there are no obvious cracks at the connection interface between the enlarged section and existing beam section of all specimens, which indicates that the bonding performance between the enlarged section and existing beam section is reliable. After strengthening, the enlarged section and existing beam section are commonly worked together and deformed harmoniously.

### 3.2. Load–Displacement Curves

**Figure 10:**
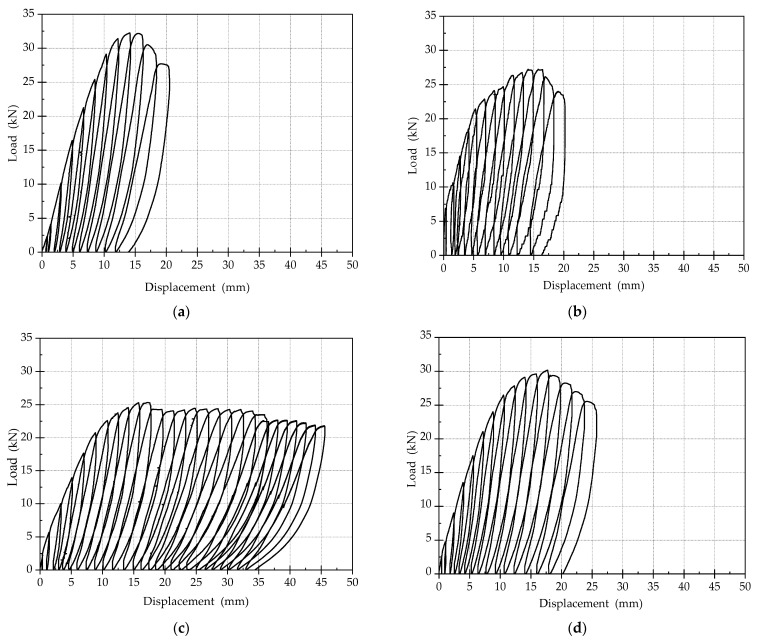
Load displacement curves (**a**) SJ-1; (**b**) SJ-2; (**c**) SJ-3; (**d**) SJ-4.

The load–displacement curves for all the specimens are shown in Figure 10. By comprehensively comparing the load–displacement curves of the four specimens, it can be seen that:

(1) Comparing the number of loading cycles of the load–displacement curves of the four specimen, it can be found that the number of loading cycles of beams strengthened by ECC is more than that of beams strengthened by concrete, indicating that strengthening with ECC can significantly improve the ductility of specimens. Among all tested members, the ductility of SJ-3 is the best, followed by SJ-4, and the ductility of SJ-1 and SJ-2 are the worst.
(2) The ultimate bearing capacities of SJ-1 and SJ-4 are higher than that of the other two specimens, because both SJ-1 and SJ-4 are reinforced by steel bars. The total tensile capacity of steel reinforcements is slightly greater than that of SMA bars, and the bond strength of ribbed steel bar in concrete or ECC is better than that of the SMA bar.
(3) The residual deformation of SJ-2 and SJ-3 after unloading is smaller than the other two specimens. However, the self-recovery performances of SJ-2 and SJ-3 are still not obvious, and the super-elasticity of SMA is not significantly displayed while strengthening beams.

### 3.3. Skeleton Curves

**Figure 11:**
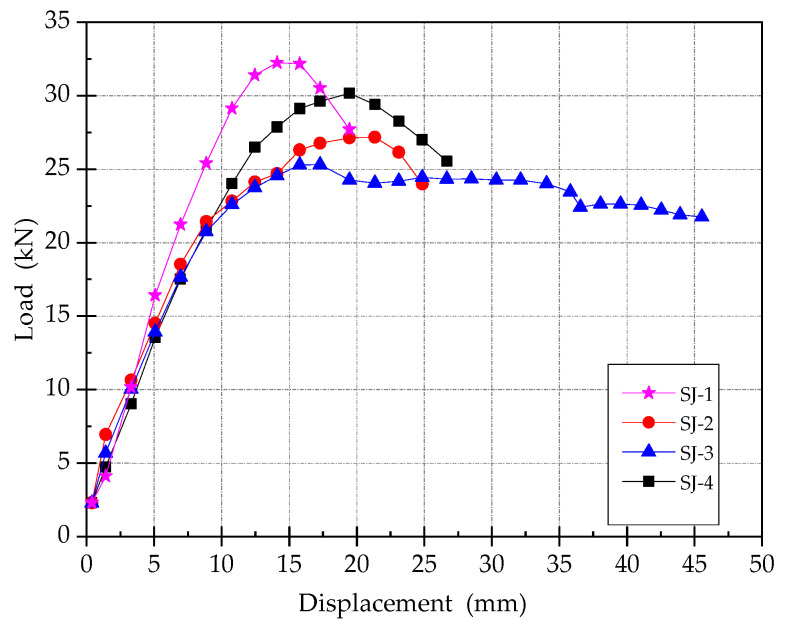
Skeleton curves.

The skeleton curves of 4 specimens are shown in Figure 11. By comprehensively comparing those skeleton curves, it can be seen that:

(1) All the test processes of the four specimens have gone through three stages, respectively, which are elastic stage, elastic-plastic stage, and failure stage.
(2) Replacing the steel bars in the concrete enlarged section by SMA bars with equivalent strength, the ultimate bearing capacity of the beam reduces by 15.5%. If the concrete is replaced by ECC, the ultimate bearing capacity decreases by about 6%. However, the number of loading cycles of ECC specimen is significantly more that of the concrete specimen, which shows that ECC can improve the ductility of specimens.
(3) After reaching the ultimate bearing capacity, the bearing capacity of SJ-3 decreases significantly slower than that of the other three members, followed by SJ-4, and SJ-1 decreases the fastest. It indicates that the ductility of SJ-3 and SJ-4 is significantly improved while being strengthened by ECC.

### 3.4. Maximum Crack Width

**Figure 12:**
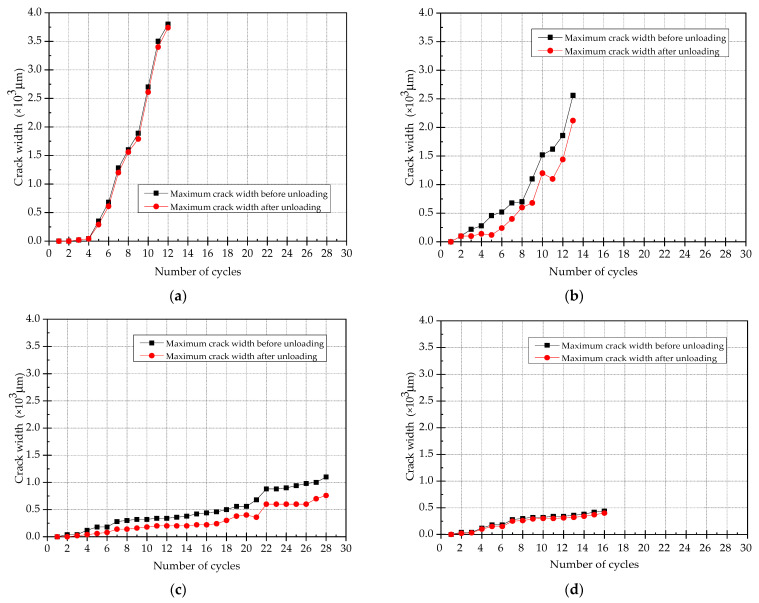
The curves of maximum fracture width under loading and unloading. (**a**) SJ-1; (**b**) SJ-2; (**c**) SJ-3; (**d**) SJ-4.

The curves of maximum crack width distribution during loading and unloading are shown in Figure 12. The following conclusions can be drawn from the comparison between those curves:

(1) As the number of loading cycle increases, the cracks continue to develop and the crack width becomes wider. After unloading, the values of maximum crack width for all specimens reduce, but the reductions are quite different.
(2) By comparing the maximum crack width before and after unloading, it can be seen that the reduction of the maximum crack width of SJ-1 and SJ-4 after unloading is very small and can be basically ignored. The maximum crack width of SJ-2 and SJ-3 decreases obviously after unloading, the decreasing rates are 19.2% and 31.8%, which indicates that the self-recovery performances of specimens can be improved while strengthening with SMA. Due to the use of plain SMA bars as the reinforcement, the bond strength between SMA bars and concrete/ECC is small. Therefore, the super-elasticity of SMA cannot be fully utilized in the deformation process of the specimen. That is the reason why the specimens of SJ-2 and SJ-3 can only be partially recovered after unloading.
(3) By comparing the values of maximum crack width for all specimens, the maximum crack widths of SJ-3 and SJ-4 are much smaller than the other 2 specimens, which are less than 500μm before the 16th loading cycle. Due to its good ductility, the specimen of SJ-3 can be continuously loaded until the vertical displacement in mid-span reaches 42 mm (the 28th loading cycle). The maximum crack width of SJ-3 at this time is only 1078 μm, which is still less than the maximum crack widths of SJ-1 and SJ-2. It proves that using ECC as reinforcement layer can effectively control the development of crack width in the tensile zone of the beam section.

### 3.5. Number of Cracks

**Figure 13:**
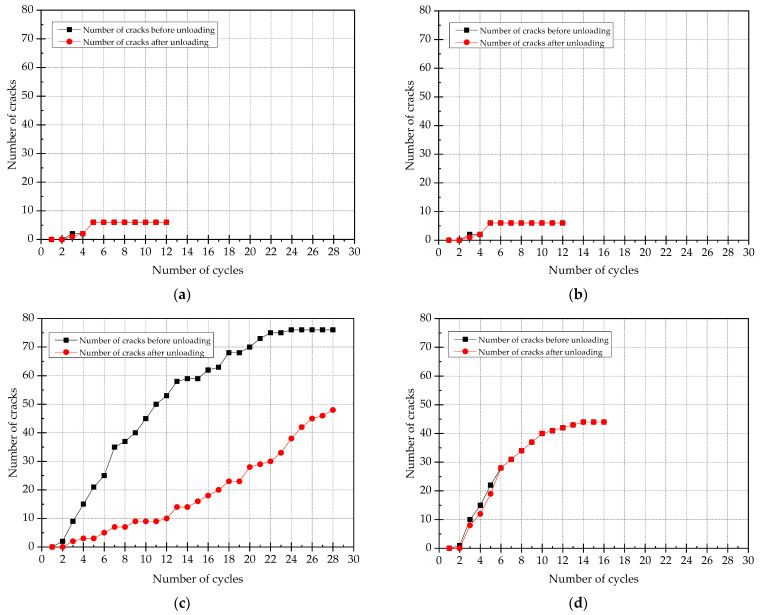
The curves of number of cracks under loading and unloading. (**a**) SJ-1; (**b**) SJ-2; (**c**) SJ-3; (**d**) SJ-4.

The curves of number of cracks for each specimen is shown in Figure 13. By comparing the results of data analysis, it can be found that:

(1) For specimens strengthened with ECC, the number of cracks in the tensile area of beam section is significantly more than that of concrete specimens. With the increase of loading cycles, ECC strengthened specimens will quickly produce new cracks, but the crack width does not increase significantly. However, after the cracking of concrete specimens, the crack width increases with the increasing load in order to form obvious main cracks, and the number of cracks does not increase significantly in the later cycles of loading.
(2) By comparing the curves of number of cracks of SJ-2 and SJ-3, it can be seen that the number of cracks of SJ-2 does not reduce significantly after unloading, but the number of cracks of SJ-2 decreases significantly after unloading. It indicates that the development of fine cracks is conducive to the shape memory effect and super-elasticity of SMA. The self-recovery performance of beams can be better realized by strengthening beams with SMA reinforced ECC layer.
(3) By comparing the curves of number of cracks of SJ-1 and SJ-2, two curves are basically the same, and the number of cracks after unloading does not decrease. This is because the bonding performance between SMA and concrete is poor, so the super-elasticity of SMA is not effective under this situation.

### 3.6. Mid-Span Deflection

**Figure 14:**
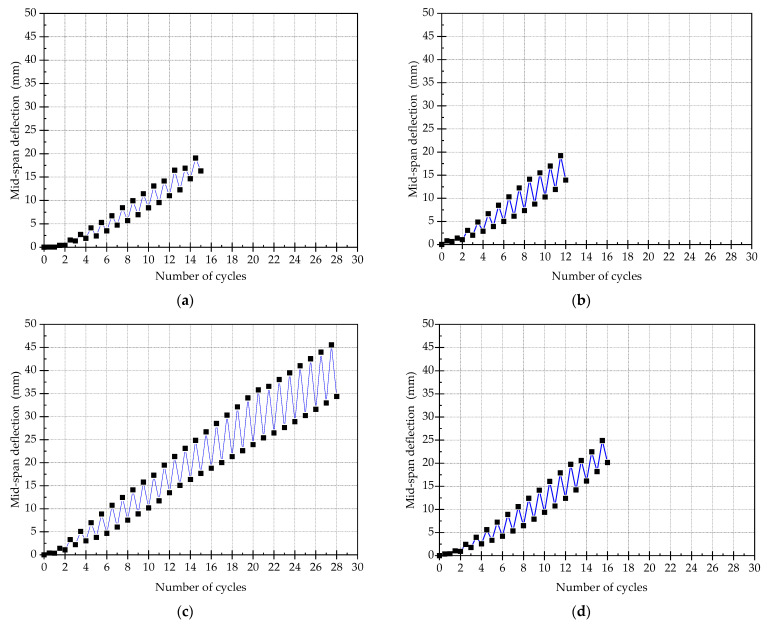
The curves of mid-span deflection. (**a**) SJ-1; (**b**) SJ-2; (**c**) SJ-3; (**d**) SJ-4.

The curves of mid-span deflection for all the specimens are shown in Figure 14. By comparing these curves, it can be seen that as the loading cycle increases, the mid-span deflection of the specimen increases linearly. After unloading, the mid-span deflections of the four specimens recover, while the self-recovery performance of SJ-3 is obviously the best, followed by SJ-2 and SJ-4, and the value of recovered mid-span deflection for SJ-1 is the minimum. The maximum recovery rates for all components are 28.4% for SJ-1, 42.7% for SJ-2, 26.1% for SJ-3, and 27.1% for SJ-4, respectively. It indicates that: (1) The mid-span deflection of the strengthened beam can be actively recovered by use of shape memory effect and super-elasticity of SMA; (2) The self-recovered value of mid span deflection of beams strengthened with ECC is obviously better than that of concrete beams, which proves that the failure mode of fine cracks of ECC can provide good conditions for self-recovery of the strengthened beams after unloading; (3) The recovery value of mid span deflection of ECC members is large, but due to the low stiffness of ECC members, the recovery rate is less than that of concrete members; (4) The recovery value of mid span deflection of ECC members is large, but due to the low stiffness of ECC members, the recovery rate is less than that of concrete members

### 3.7. Energy Consumption Capacity

**Figure 15:**
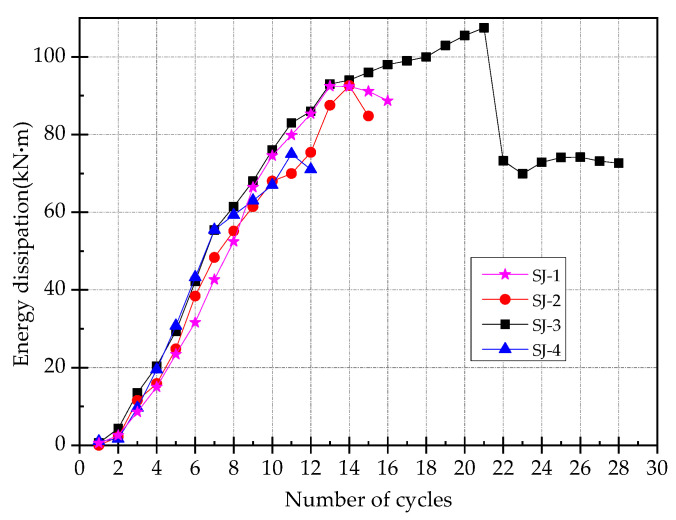
The curves of energy consumption capacity.

The energy consumption capacity of the specimen can be determined by the area enveloped by the load–displacement curve of each level of loading. The curves of energy consumption capacity for all the specimens are shown in Figure 15. It can be seen that the energy consumption capacity of the specimen SJ-3 is the highest. Before the 22nd cycle, the energy dissipation capacity is mainly borne by ECC, which is in the strain-hardening stage. At the 23rd cycle, the ECC layer began to failure, the energy consumption capacity is mainly borne by SMA at this moment, and it is significantly reduced. Before the specimen is completely failed, the values of energy consumption for all specimens are basically the same. This is because the steel bars inside the original beam section are still retained after strengthening of the beams. With the reinforcement of the enlarged section, all the reinforcements at the bottom section of the beam do not yield obviously during the tests. Therefore, there is no obvious difference in the energy consumption capacity between each specimen.

### 3.8. Mechanical Performance of Reinforcements

**Figure 16:**
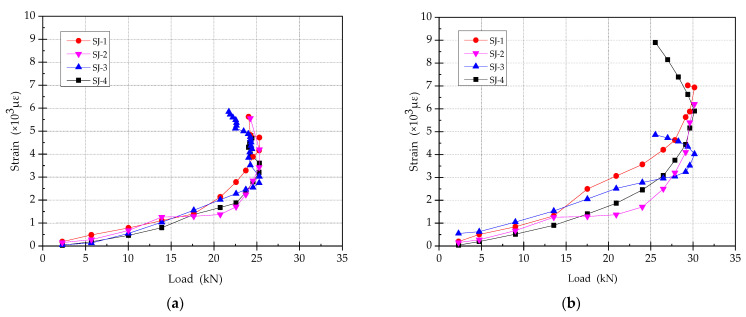
The load–strain relationship between the reinforcement. (**a**) The steel bar in existing beam section; (**b**) The reinforcements in enlarged section.

Figure 16 shows the load–strain relationship between the reinforcement in the enlarged section and the steel bar in original section. It can be concluded from the analysis of those results that: (1) The strain development of the original reinforcements is basically similar, which shows that all the strengthening methods of the specimens can give full play to the material properties of the original reinforcements, and the mechanical performances of the strengthened specimens are good. (2) The strain development of reinforcements in the enlarged section is also relatively stable in the elastic stage. With the increase of load, the reinforcement of SJ-1 yields first, followed by SJ-2 and SJ-4, and the reinforcement of SJ-3 yields last. This shows that ECC in the enlarged section can bear part of the tensile force, which makes the reinforcements in the tensile area yield later so as to improve its recovery ability.

## 4. Flexural Capacity Formula

### 4.1. Basic Formula

**Figure 17:**
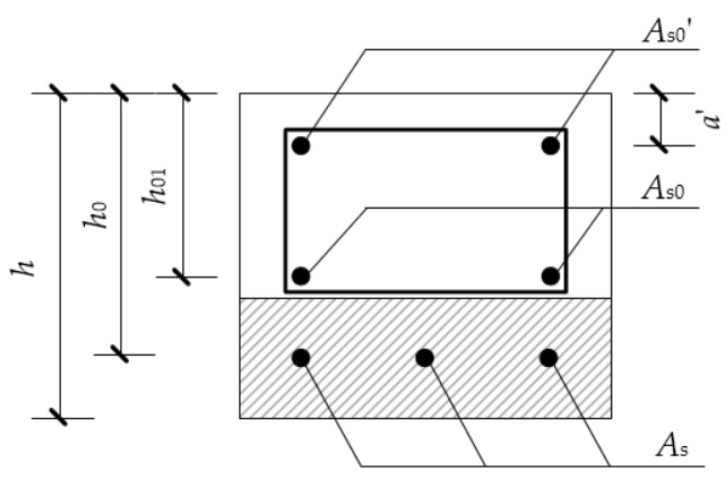
Dimensions of strengthened beam.

According to the “Code for design of strengthening concrete structure” (GB50367-2013) [45], the flexural bearing capacity of the existing beam section should be determined according to Formula (1):

(1) $$M\leq \alpha_{s}f_{y}A_{s}(h_{0}-\frac{x}{2})+f_{y0}A_{s0}(h_{01}-\frac{x}{2})+f_{y0}^{’}A_{s0}^{’}(\frac{x}{2}-a^{’})$$

where:

*M*—Design value of bending moment after strengthening of member (kN·m)
*α*_s_—Strength utilization factor of reinforcements in enlarged section, taken as *α*_s_ = 0.9
*f*_y_—Design value of tensile strength of reinforcements in enlarged section (N/mm^2^)
*A*_s_—The cross-sectional area of the reinforcements in enlarged section (mm^2^), as shown in Figure 17.
*h*_0_, *h*_01_—Effective height of section after strengthening and before strengthening (mm), as shown in Figure 17.
*x*—Height of compression zone of the section concrete (mm)
*f*_y0_, *f’*_y0_—Design value of tensile and compressive strength of steel bars in existing structure member (N/mm^2^)
*A*_s0_, *A’*_s0_—The cross-sectional area of the tensile reinforcements and compressive reinforcements (mm^2^), as shown in Figure 17.
*a*’—The distance from the resultant force point of longitudinal compressive reinforcements to the edge of compression zone of the beam (mm), as shown in Figure 17.

### 4.2. Flexural Capacity of ECC Reinforced Beams

**Figure 18:**
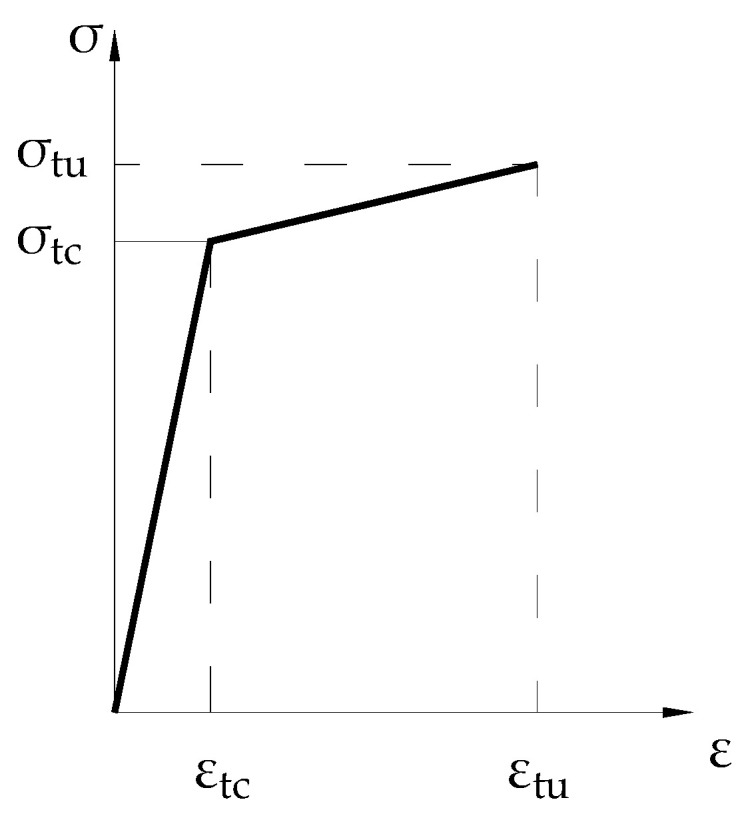
ECC tensile stress–strain curve.

Through the verification of the test results, it is found that when ECC is used to strengthen the tensile zone of the beam, due to the excellent tensile performance of ECC, the contribution of ECC to the bending capacity of the strengthened beam must be considered. Therefore, formula 1 needs to be revised. The revised formula is shown as Formulas (2) and (3).

(2) $$M\leq \alpha_{s}f_{y}A_{s}(h_{0}-\frac{x}{2})+f_{y0}A_{s0}(h_{01}-\frac{x}{2})+f_{y0}^{’}A_{s0}^{’}(\frac{x}{2}-a^{’})+F_{t,ecc}\times (h-\frac{x}{2}-\frac{h_{1}}{2})$$

(3) $$F_{t,ecc}=f_{t,ecc}\times b\times h_{1}$$

where:

*f*_t,ecc_—Equivalent strength of ECC (N/mm^2^), Use value of σ_tc_ in the tensile stress–strain relationship curve of the ECC shown in Figure 18 [46]
*b*—Section width (mm)
*h*_1_—The height of enlarged section strengthened by ECC (mm), *h*_1_ = 40 mm in these tests

### 4.3. Verification of the Revised Formula

By using the parameters of material properties in Table 4 and Table 5, the theoretical value of flexural bearing capacity of each specimen can be calculated through the revised formula.

The theoretical values and the experimental value of flexural bearing capacity for all 4 specimens are shown in Table 6, where *M*_cu_ is the calculated theoretical value of the bearing capacity of the beam strengthening with increasing section area, *M*_tu_ is the experimental value of the bearing capacity of the beam strengthening with increasing section area, which can be determined by the loading when the reinforcements are yielded, and *M*_cu_/*M*_tu_ is the ratio of the theoretical value to the experimental value.

It can be seen from Table 6 that the bending capacity calculated by Formula (2) for all the specimens are in good agreement with the test value, and the errors are all within 10%, indicating that the accuracy of the revised formula can be guaranteed. The values of *M*_cu_ are always less than the value of *M*_tu_, representing that the theoretical values calculated by Formula (2) are much safer compared with the actual value, and the revised formula can be well applied to beam strengthening with increasing section of ECC.

## 5. Conclusions

(1) The effects of heat treatment, strain amplitude, and number of loading cycle on the mechanical properties of SMA were studied. The results show that the mechanical properties of SMA can be improved by heat treatment significantly; the stability of mechanical properties of SMA can be significantly improved by increasing the strain amplitude and loading cycle.
(2) The reinforced concrete beam strengthened with increasing section of ECC have good toughness, the cracking characteristics of the strengthened beam is the fine cracks when it fails. The strengthened beam can continue to bear the load beyond the ultimate bearing capacity, and the bearing capacity decreases slowly, indicating that the beam strengthening with increasing section of ECC has good energy dissipation capacity.
(3) The total tensile capacity of steel reinforcements is slightly greater than that of SMA bars, and the bond strength of ribbed steel bar in concrete or ECC is better than that of SMA bar. Therefore, the bearing capacity of specimens strengthened with steel is better than that of SMA bar.
(4) The crack width, number of cracks, and recovery performance of concrete beams strengthened with SMA bars are better than those of ordinary reinforced concrete beams. In order to give full play to shape memory effect and super-elasticity of SMA, the bond strength between SMA and concrete/ECC should be improved. The effect of temperature on the material properties of SMA cannot be ignored.
(5) The combination of SMA and ECC gives full play to their own respective advantages, respectively. ECC provides good toughness and cracking characteristics, and SMA provides excellent recovery ability. These two materials working together can significantly improve the reliability of the structure.
(6) Based on the design formula of bending capacity recommended by the design code and considering the tensile capacity provided by ECC in the strengthened section, a revised design formula for the bending bearing capacity of RC beams strengthened with increasing section of ECC is proposed. The revised design formula are well demonstrated by the test results, indicating that the revised formula can be well applied to the beam strengthening with increasing section of ECC.

The purpose of this paper is to reveal the bending capacity, failure mode, and self-recovery capacity of concrete beams strengthened with SMA/ECC enlarged section based on full-scale beam specimens with small dimensions. In the follow-up research, the influence of different design parameters, such as section size, flexure reinforcement ratio, and material strength, etc., on the flexural performance of concrete beams strengthened with SMA/ECC enlarged section will be further analyzed theoretically and experimentally to improve the design principle of the strengthening method and provide a theoretical basis for design of strengthening works.

## Figures and Tables

**Table 1:** The design parameters of specimens.

Serial Number of Specimen	Strengthening Material	Section Size (mm)	Beam Length (mm)	Reinforcement	Reinforcement Diameter
SJ-1	Steel-concrete	120 × 110	1000	2 HRB355 steel bars	6 mm
SJ-2	SMA-concrete	120 × 110	1000	3 SMA bars	5.5 mm
SJ-3	SMA-ECC	120 × 110	1000	3 SMA bars	5.5 mm
SJ-4	Steel-ECC	120 × 110	1000	2 HRB355 steel bars	6 mm
SJ-5	Steel-concrete	120 × 110	1000	2 HRB355 steel bars	5.5 mm
SJ-6	SMA-ECC	120 × 110	1000	3 SMA bars	5.5 mm

**Table 2:** Mix proportion of ECC.

Element	Cement	Water	Fly Ash	Fine Sand	Admixture	PVA Fiber
Proportion	1	1.43	1.43	0.86	0.18	2%

**Table 3:** Tensile test results of ECC specimens.

Specimen Number	First Group	Second Group	The Third Group	Average Value
Tensile strength (MPa)	4.26	3.89	3.46	3.87

**Table 4:** Yield strength of longitudinal reinforcement.

Material	SMA	Tensile Longitudinal Bar	Compressed Longitudinal Bar
Yield Strength (MPa)	296.17	397.17	397.17

**Table 5:** Concrete strength.

Material	Concrete	ECC
Compressive strength (MPa)	17.48	18,021
Tensile strength (MPa)	-	5.1

**Table 6:** The theoretical values and the experimental values of bending capacity for the strengthened beams.

Specimen Number	Reinforcement Material	*M*_cu_ (kN·m)	*M*_tu_ (kN·m)	*M*_cu_/*M*_tu_
SJ-1	Steel-Concrete	2.75	2.96	0.92
SJ-2	SMA-Concrete	2.51	2.76	0.91
SJ-3	SMA-ECC	2.51	2.78	0.90
SJ-4	Steel-ECC	2.75	2.93	0.94
